# Anxiety and Depression Among Astana Reinfected Patients at 1-, 3-, and 6-Month Follow-Up in the Post-COVID Center

**DOI:** 10.1155/carj/5596465

**Published:** 2025-02-24

**Authors:** Makhabbat Bekbossynova, Ainur Tauekelova, Zhanar Kalila, Aliya Sailybayeva, Sadyk Khamitov, Zhansaya Oralbekova

**Affiliations:** ^1^Clinical Academic Department of Cardiology, Heart Center (Corporate Fund “University Medical Center”), Astana, Kazakhstan; ^2^Research Department, Heart Center (Corporate Fund “University Medical Center”), Astana, Kazakhstan; ^3^Health and Well-Being Department, Nazarbayev University, Astana, Kazakhstan

**Keywords:** anxiety, astana, COVID-19, depression, post-COVID

## Abstract

We present the findings from an evaluation of 144 survivors who experienced post-COVID-19 complications or reinfection. The assessment was conducted at 1, 3, and 6 months following their discharge from an intensive post-COVID care center. The evaluation encompassed a comprehensive analysis of clinical life-critical indicators and mental health states. Based on lung CT scans, pneumonia was identified in 73% of the patients, categorized into four severity groups according to their health conditions: mild (26%), moderate (57%), severe (14%), and extremely severe (3%). Among the extremely severe cases, two patients succumbed to the illness. Self-reported fatigue during the acute phase was prevalent among 79% of participants, which increased to 91% at 1 month, decreased to 64% at 3 months, and further decreased to 56% at 6 months. The vital signs of patients, including systolic and diastolic blood pressure, oxygen saturation, body temperature, respiratory rate, and heart rate, gradually normalized over time. Anxiety and depression symptoms persisted in 17% and 21% of patients, respectively, up to the 6-month mark; even though during the acute phase, these figures were 15% and 13%. The limitations of this study include issues related to sample representation and the exclusion of hypertension data, which affect the overall balance of its findings.

**Trial Registration:** ClinicalTrials.gov identifier: NCT04987853

## 1. Introduction

Patients affected by severe acute respiratory syndrome coronavirus 2 (SARS-CoV-2) often grapple with persistent respiratory, physical, and mental health challenges that extend well beyond their initial hospitalization, spanning from 1 to 50 months [1]. Similarly, individuals who experience reinfection with SARS-CoV-2 deal with lasting effects [2]. Notably, there is a shortage of comprehensive studies examining the vital signs and mental health of reinfected patients who have undergone multiple discharges from the intensive care unit (ICU). Our objective was to delineate the long-term clinical outcomes, assess the severity of fatigue, explore psychiatric disorders, and evaluate the quality of life among patients who had been reinfected with COVID-19 and previously developed acute respiratory distress syndrome (ARDS). This investigation spans the periods of 1, 3, and 6 months following the acute phase, focusing on individuals who survived hospital discharge.

## 2. Methods

In this prospective follow-up study, we examined 144 adult patients (aged 18 years and older) who had previously been hospitalized with confirmed SARS-CoV-2 infection, verified by polymerase chain reaction (PCR). These individuals were discharged alive from the ICUs of 21 medical facilities between January 27, 2022, and July 31, 2022. Our focus was on investigating the persistence of previous COVID-19 infection and related symptoms. Subsequent follow-up assessments were after 1, 3, and 6 months and took place at the National Research Cardiac Surgery Center in Astana, Kazakhstan. To be included, patients needed to have a confirmed positive diagnosis of SARS-CoV-2 reinfection with PCR, reside in Astana city, and should have been previously hospitalized in COVID-19 treatment centers, all while being aged 18 years or older. Those who declined to undergo the diagnostic procedures outlined in the study protocol were excluded from participation. Before the study commenced, all participants provided written informed consent to be part of the research. Our research primarily focuses on Astana, which may limit the generalizability of the results, and there is also the absence of hypertension data, which may significantly impact the assessment of mental health outcomes in our study; therefore, future studies should explore other regions of Kazakhstan and include hypertension data, allowing for a more thorough understanding of the interplay between these health conditions to enhance the breadth and applicability of the findings.

### 2.1. Follow-Up Protocol

The follow-up protocol includes an initial in-person doctor's checkup, followed by the assessment of vital signs. Subsequently, participants are required to respond to questionnaires and undergo a CT scan after six months. Additional information can be found on the clinical trial's website. In the acute phase and after 1, 3, and 6 months, patients were assessed through structured in-person checkups at the center accompanied by a doctor. During the entire visit of patients, we collected demographic data, comorbidities, WHO severity stage [3], vaccination information, and smoking status. In addition, we collected the lung computerized tomography (CT) scan results of patients from the previous ICU.

In the acute phase and after 1, 3, and 6 months, we conducted a standardized local clinical assessment, emphasizing the vital physiological functions, mental health evaluated questionnaires, and self-reported symptoms. Vital signs [4] such as systolic blood pressure, diastolic blood pressure, oxygen saturation, body temperature, respiratory rate, and heart rate were collected and measured by the triage method. Also, it is crucial to emphasize the importance of incorporating diverse geographical and demographic factors, as this acknowledgment helps clarify the limitations inherent in our findings. Most self-reported fatigue was assessed by The Chalder Fatigue Scale (CFQ 11). We used CFQ 11 to measure the extent and severity of fatigue within both clinical and nonclinical epidemiological populations. Instead of utilizing the subscales for physical and psychological fatigue, this approach involves assigning a global binary fatigue score ranging from 0 to 11, where a score of 3 or less indicates the absence of fatigue, while scores of 4 or more signify “severe fatigue,” as established by the authors [5]. Also, we used the EuroQol-visual analog scales (EQ-VAS) to collect information about patients' global assessment of their health conditions in a range of 0 (*the worst possible health*) and 100 (*the best possible health*). To assess anxiety and depression, we used the Hospital Anxiety and Depression Scale (HADS); if the score fell above or equal to 8, each was deemed abnormal.

The study sample included all eligible survivors who received treatment for positive PCR results during the study period without doing an a priori assessment of the sample size. We summarized numeric variables by the mean and standard deviation or median and interquartile range (IQR) (Q1–Q3), whereas we used counts and percentages for quantitative variables. For statistical comparison between four groups of the acute phase, after one month, after three months, and after six months of continuous variables, we used analysis of variance (ANOVA). Fisher's exact test was used to assess categorical variables. In all tests, *p* values less than 0.05 were considered statistically significant. We conducted the statistical analysis using R-4.3.0 and SPSS software.

## 3. Results

In total, 358 patients were admitted to the post-COVID ICU. The 308 patients showed their eagerness to participate in the long-term follow-up; however, at that time, only 144 patients were reinfected, had positive PCR, and were discharged alive from ICU with ARDS previously. The mean age of the participants was 59 years (SD 13), and the body mass index (BMI) was 29.05 (mean), 5.88 (SD). CT scan results of patients from the previous ICU shows that 73% of the patients were diagnosed with pneumonia. The demographic and clinical data of the acute phase are shown in Table 1. The obesity classification based on the clinical staging system for obesity [6]. 6 vaccines were approved for use in Kazakhstan, which are Pfizer/BioNTech Comirnaty, Gamaleya Sputnik Light, Gamaleya Sputnik V, Research Institute for Biological Safety Problems (RIBSP) QazVac, Sinopharm (Beijing) Covilo, and Sinovac–CoronaVac [7].

After one month of discharge from the post-COVID center, 114 (79%) patients continued follow-up, 2 patients died, and 28 patients lost follow-up. After 3 months, 107 (74%) patients came and 7 participants lost follow-up. After 6 months, 106 (73.6%) patients revisited the doctor three times and only 1 patient lost follow-up. A follow-up CT scan was conducted 6 months later, revealing that the prevalent observed abnormalities included fibrotic changes in bronchitis 36% (*n* = 38) of cases and mediastinal lymphadenopathy in 10% (*n* = 9).

**Figure 1:**
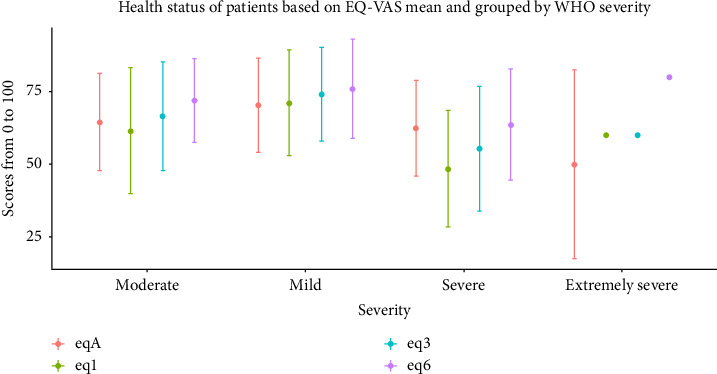
Health status of patients over time and severity.

At one month, the CFQ 11 showed a median of 6 (IQR 11–2) which means the level of tiredness increased later (Table 2). The subjective health experience EQ-VAS (Figure 1) at the acute phase of reinfection median was 67.5 (IQR 80–50) having a marked advancement over time (Table 2). The heart rate at the acute episode median was 84 bpm (IQR 93–76 bpm) with a sustained substantial improvement.

The HADS is a widely used questionnaire designed to assess the levels of anxiety and depression in hospital settings. Comprising 14 items—seven focused on anxiety and seven on depression—HADS offers a brief yet effective means of screening for these conditions. Its relevance lies in its ability to distinguish between anxiety and depression symptoms, making it particularly useful in clinical settings where comorbidity is common. Compared to other tools, such as the Beck Depression Inventory or the State-Trait Anxiety Inventory, HADS is less time-consuming and can be easily administered, providing quick insights into patients' emotional states. Based on the HADS sum, participants' mental health impairments remained the same over time. Anxiety was found among 15% (*n* = 22) of patients at the acute episode with a sharp increase at 3 months to 26% (*n* = 28), while at 1 and 6 months it was 12% (*n* = 14) and 17% (*n* = 18) (*p* = 0.029), respectively. The anxiety had increased at 3 months, and the depression had increased at 6 months. At acute phase and at 1, 3, and 6 months, depression levels were 13% (*n* = 19), 12% (*n* = 14), 18% (*n* = 19), and 21% (*n* = 22) (*p* < 0.029), respectively.

## 4. Discussion

In this extensive follow-up analysis of patients who were admitted with COVID-19 and later developed ARDS, we discovered that after 1 and 3 months of reinfection, 91% and 64% of patients self-reported severe fatigue, and 57% and 48% reported problems with memory in the CFQ 11. In the one-time study on mental health on pandemic period in Kazakhstan where 12.01% reported depressive symptoms and 8.38% anxiety; however, the severity of infection and follow-up were not published [8]. Our findings showed similar fatigue severity levels with Bangladesh COVID-19 survivors which was 62.9% suffers after two months of ICU discharge [9].

Compared with the Netherlands cohort study, fewer people in our follow-up suffered mental illnesses after 3 months of hospitalization. Anxiety (26% vs. 33.9%), and depression (18% vs. 50%) after 6 months scored more than or equal to 8 in HADS (anxiety [17% vs. 29%] and depression [21% vs. 41.9%]) [10]. The notable difference in mental health conditions may be related to the average population age, which was 59 in our study and 50 in the previous study [10], and age might not only be the factor responsible for the difference in prevalence of anxiety and depression between Europe and Kazakhstan. Culturally, the Netherlands has a well-established support system for mental health, characterized by greater public awareness and reduced stigma surrounding mental health issues. This supportive environment may encourage individuals to seek help earlier and more frequently, leading to better overall mental health outcomes compared to populations in regions with less access to mental health resources or where stigma is more prevalent. Socioeconomically, the Dutch cohort benefits from a robust healthcare system, high living standards, and greater access to education and employment opportunities. In contrast, our cohort may experience economic challenges, higher unemployment rates, and limited access to healthcare services, which can exacerbate stress and negatively impact mental health. Additionally, differences in social support networks, community engagement, and coping mechanisms can further contribute to the observed disparities. By considering these cultural and socioeconomic factors, we can better interpret our results and highlight the need for targeted interventions that address these underlying issues, ultimately aiming to improve mental health outcomes in populations with different contexts. Future research should explore these dimensions further to deepen our understanding of the complexities involved. This study demonstrated the necessity of taking into consideration the age difference of patients during the follow-up and providing care. Respiratory rate was similarly normalized after three months from the acute phase as in the study of Italian COVID-19 survivors with a median age of 61.8 [11].

A significant limitation of our study is the absence of a control group, which poses challenges in attributing observed changes in mental health solely to reinfection. Without a control group for comparison, it becomes difficult to isolate the effects of reinfection from other potential influencing factors. To enhance the robustness of future research, we propose the inclusion of control groups, which would allow for more accurate assessments of mental health outcomes and a clearer understanding of the relationship between reinfection and psychological well-being. The limitation of the study is the absence of information regarding individuals with hypertension and those on antihypertensive medications. The strength of our study is the in-person follow-up at each stage of the research, despite the second or third wave of COVID-19. It was an essential support for the patients. Also, participants even if taken care of in one post-COVID center, they had previously been assessed in multiple ICUs. The research is still going, and further findings of the project will be published.

To sum up, at this stage of the mental health, investigation taking into account the vital signs of the patients before giving mental health questionnaires was important. The overall quality of life among patients improved over time even in the different severity groups.

## Figures and Tables

**Table 1:** Demographic and clinical characteristics, and comorbidities of patients during the acute COVID-19 episode in the post-COVID center.

		Count (*n*)	Proportion/percent
Gender	Female	87	0.6/60
	Male	57	0.4/40

Severity	Mild	37	0.26/26
	Moderate	82	0.57/57
	Severe	20	0.14/14
	Extremely severe	5	0.03/3

Obesity (10)	No	92	0.64/64
	Stage 0	2	0.014/1.4
	Stage 1	28	0.19/19
	Stage 2	14	0.1/10
	Stage 3	8	0.056/5.6

Diabetes	Diabetes	22	0.15/15
	Prediabetes	6	0.04/4
	No	116	0.81/81

Vaccine	Unvaccinated	87	0.60/60
	Vaccinated	57	0.40/40

Smoking	No	129	0.90/90
	Yes	15	0.1/10

Pneumonia	Yes	105	0.73/73
	No	39	0.27/27

**Table 2:** Vital signs and mental signs of survivors of COVID-19 after discharge from the post-COVID center.

	Acute (*n* = 144)	1 month (*n* = 114)	3 months (*n* = 107)	6 months (*n* = 106)	*p* value
Systolic blood pressure (mmHg), median (IQR)	120 (130–118.8)	130 (140–120)	120 (130–115.5)	120 (130–120)	0.0341
Diastolic blood pressure (mmHg), median (IQR)	80 (90–80)	80 (80–80)	80 (80–80)	80 (80–80)	0.378
Oxygen saturation (SpO2) (% predicted), median (IQR)	97 (98–94)	97 (98–96)	97 (98–97)	97 (98–96)	NA
Body temperature (degrees Celsius), median (IQR)	38 (38.45–37.2)	36.1 (36.3–36.0)	36.2 (36.4–36.0)	36.3 (36.4–36.2)	< 0.01
Respiratory rate (breaths per minute), median (IQR)	20 (23–20)	18 (18–18)	18 (18.04–18)	18 (18–18)	< 0.01
Heart rate (bpm), median (IQR)	84 (93–76)	76 (80–70)	74 (78–70)	70 (76–66)	< 0.01
EQ Visual Analog Scale (EQ VAS) (% predicted), median (IQR)	67.5 (80–50)	70 (80–40)	70 (80–50)	75 (80–60)	0.00166
The Chalder Fatigue Scale (CFQ 11), median (IQR)	5.5 (8–2)	6 (11–2)	4 (9–0)	3 (7–0)	0.000127
Hospital Anxiety and Depression Scale (HADS) (sum), median (IQR)	8 (12.25–4)	7 (11–4)	9 (15–4.25)	7 (13–3)	0.017

## Data Availability

The data used in this study are available at https://clinicaltrials.gov/ct2/show/NCT04987853?cond=covid&cntry=KZ&draw=2&rank=8.
